# Chronic stroke patients show early and robust improvements in muscle and functional performance in response to eccentric-overload flywheel resistance training: a pilot study

**DOI:** 10.1186/1743-0003-11-150

**Published:** 2014-10-30

**Authors:** Rodrigo Fernandez-Gonzalo, Catarina Nissemark, Birgitta Åslund, Per A Tesch, Peter Sojka

**Affiliations:** Department of Physiology & Pharmacology, Karolinska Institutet, Sweden; Östersund Rehabcentrum Remonthagen, Östersund, Sweden; Department of Health Sciences, Mid Sweden University, Östersund, Sweden

**Keywords:** Balance, Bilateral asymmetry, Muscle strength, Neuro-rehabilitation

## Abstract

**Background:**

Resistance exercise comprising eccentric (ECC) muscle actions enhances muscle strength and function to aid stroke patients in conducting daily tasks. The purpose of this study was to assess the efficacy of a novel ECC-overload flywheel resistance exercise paradigm to induce muscle and functional performance adaptations in chronic stroke patients.

**Methods:**

Twelve patients (~8 years after stroke onset) performed 4 sets of 7 coupled concentric (CON) and ECC actions using the affected limb on a flywheel leg press (LP) device twice weekly for 8 weeks. Maximal CON and ECC isokinetic torque at 30, 60 and 90°/s, isometric knee extension and LP force, and CON and ECC peak power in LP were measured before and after training. Balance (Berg Balance Scale, BBS), gait (6-Min Walk test, 6MWT; Timed-Up-and-Go, TUG), functional performance (30-s Chair-Stand Test, 30CST), spasticity (Modified Ashworth Scale) and perceived participation (Stroke Impact Scale, SIS) were also determined.

**Results:**

CON and ECC peak power increased in both the trained affected (34 and 44%; P < 0.01), and the untrained, non-affected leg (25 and 34%; P < 0.02). Power gains were greater (P = 0.008) for ECC than CON actions. ECC isokinetic torque at 60 and 90°/s increased in the affected leg (P < 0.04). The increase in isometric LP force for the trained, affected leg across tests ranged 10-20% (P < 0.05). BBS (P = 0.004), TUG (P = 0.018), 30CST (P = 0.024) and SIS (P = 0.058) scores improved after training. 6MWT and spasticity remained unchanged.

**Conclusions:**

This novel, short-term ECC-overload flywheel RE training regime emerges as a valid, safe and viable method to improve muscle function, balance, gait and functional performance in men and women suffering from chronic stroke.

**Electronic supplementary material:**

The online version of this article (doi:10.1186/1743-0003-11-150) contains supplementary material, which is available to authorized users.

## Background

Stroke is a leading cause for long-term disability[1] that often compromises muscle strength, power, balance and gait[2–5] frequently accompanied by spasticity[6, 7]. To overcome adverse neuromuscular changes and associated impairments consequent to stroke, various exercise intervention strategies have been implemented[8]. More recently, resistance exercise (RE) has become a primary target for stroke rehabilitation[9]. Indeed, RE training, challenging the more conservative approach of cautiousness, employing high-intensity muscle actions, has proven efficacy to ameliorate vital physical functions in stroke patients[10, 11] without exacerbating spasticity[12, 13]. More importantly, improvements in neuromuscular function achieved through RE training interventions appear to be carried over to long lasting benefits aiding patients in daily physical tasks[14].

For unknown reason(s) stroke patients show greater discrepancy in shortening (concentric; CON) relative to lengthening (eccentric; ECC) maximal voluntary force compared with healthy individuals[3, 4, 15]. As skeletal muscle inherently produces much less force in CON than ECC actions[16], traditional RE regimes executed by lifting and lowering weights or weight stacks, offer modest and insufficient stimulus providing the goal is maximizing neural drive and muscle activity. In support, stroke patients subjected to isokinetic RE, showed more robust neural adaptations following ECC than CON mode training[17], potentially translated into the more substantial benefits evident in functional daily activities[18]. Additionally, ECC training using the affected limb only, may elicit cross-transfer adaptations such that muscle strength and power of the non-affected limb increase as well[17]. Nevertheless, CON muscle actions should be incorporated in any rehabilitation program prescribed to stroke patients as they are equally important in daily tasks, e.g., rising from a chair or lifting a shopping bag, and obviously orchestrating with ECC actions in most activities of locomotion of coupled CON and ECC actions involving the stretch-shortening cycle.

In contrast to weight training employing constant external load, iso-inertial exercise[19] offers coupled CON and ECC actions, and maximal voluntary resistance through the full range of motion during CON actions, and if desired, brief episodes of ECC overload[19, 20]. This method, using inertial resistance provided by rotating flywheel(s) set in motion by the trainee, has shown efficacy in counteracting deleterious disuse effects producing muscle atrophy and dysfunction[21, 22]. Likewise, healthy individuals subjected to ECC-overload flywheel RE training experienced more profound increases in force and power via increased neural activation, than subjects performing conventional RE training[23, 24]. Given the unique features, ECC-overload flywheel RE emerges as an attractive approach to be offered to stroke patients.

The current study investigated the efficacy of an ECC-overload flywheel RE training challenge to enhance muscle strength and power in patients suffering from chronic stroke. We hypothesized the 8-week unilateral training intervention, using the affected lower limb, would increase force and power of both limbs, and these effects to be accompanied by improvements in balance and daily task functional performance, without provoking increased spasticity.

## Methods

### General design

**Figure 1:**
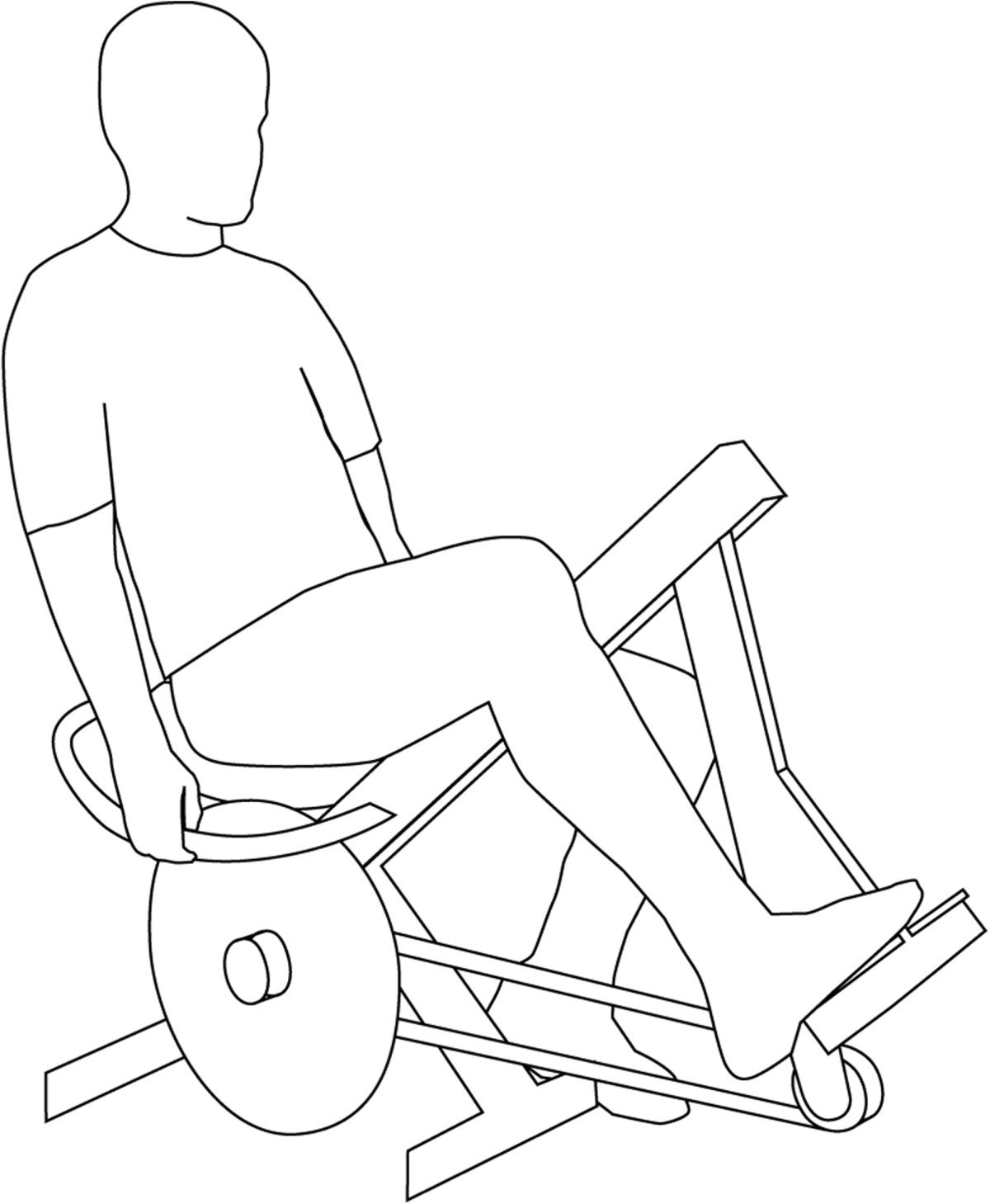
**Cartoon showing the flywheel leg press resistance exercise device for stroke patients.**

Twelve chronic stroke patients with gait deficits performed unilateral flywheel leg press (LP) RE (Figure 1) using the affected limb twice weekly for 8 weeks. Balance, gait, functional performance, and muscle function (i.e. maximal CON and ECC isokinetic torque at 30, 60 and 90°/s, maximal knee extension and LP isometric force, and CON and ECC peak power during flywheel LP exercise) were assessed before, and after the training period. Time of the day was replicated (±2 h) from pre to post training tests. Prior to any test, patients had completed 3 familiarization sessions on the LP flywheel device.

### Participants

Subjects were community dwelling and had been treated at the Östersunds Rehabcenter (Östersund Hospital, Östersund, Sweden). Inclusion criteria were (a) a history of stroke (>2 years post stroke) with unilateral motor deficits affecting gait pattern and/or speed, (b) independent walking ability with or without walking aid at least 10 m, (c) to have completed standard rehabilitation, yet not to be involved in any structured rehabilitation program for the last 6 month prior to the study and, (d) ability to perform closed-chain exercise using the prescribed LP training device. Significant psychiatric or cognitive deficits, major cardiorespiratory diseases (treated and controlled arterial hypertension were not considered exclusion criterion), chronic pain or joint affection were exclusion criteria factors. After a preliminary selection using hospital records, candidate participants were familiarized with training and test procedures, and examined by a physiotherapist and a rehabilitation medicine consultant. Possible risks and discomforts associated with the study protocols were explained and written informed consent was obtained. In particular, our pre-study assessments revealed a clear-cut risk for muscle strains and joint (mainly knee and hip) injury as the affected leg exhibited diminished joint stiffness and stability while performing unrestricted force through the entire range of motion and brief eccentric overload. Thus, a custom made device offered lateral leg support avoiding involuntary abduction of the affected limb such that the limb was stabilized and ankle, knee and hip joints positioned in the same vertical plane during leg flexion/extension. Subjects were instructed not to perform any additional strenuous lower-limb activity during the intervention. The study protocol was approved by the Regional Ethical Review Board in Umeå (No. 09-190aM; 2009-1394-31).

### Balance, gait, functional performance, spasticity and perceived participation

Tests were performed ~12 days before and after the training period by an independent physiotherapist blinded to the intervention and purpose of the study. The Berg Balance Scale (BBS;[25]) assessed balance. This test includes 14 different items to determine dynamic and static balance. Gait performance was assessed by the Timed-Up-and-Go (TUG;[26]) and the 6-Minute Walk tests (6MWT;[27]). In the TUG test, patients were instructed to rise from a chair, walk at a fast, still comfortable speed 3 m, turn around, walk back and sit down in the chair. Time to the nearest second was recorded in three trials, and the best value was used for data analysis. The 6MWT consisted of walking 6 min at a self-selected speed. The distance was recorded to the nearest meter. Functional performance was also measured by means of the 30-s Chair-Stand Test (30CST;[28]). From a seated position, patients were requested to raise from a chair to standing, and sit down as many times as possible during 30 s. The Modified Ashworth Scale[29] was used to assess spasticity of the lower limbs. Perceived participation was assessed using the Stroke Impact Scale (SIS-Patient-v.2.0[30]). Items from the SIS related to physical deficits, everyday activities and ability to move in- and outside home (domains 1, 5 and 6) were completed by the participants. Analysis of SIS was performed following Duncan et al.[30]. Briefly, mean values for domains 1, 5, and 6 were calculated (100 x (mean value of domains 1, 5 and 6 – 1)/(5 – 1)) for each patient. The greater the relative value, the fewer the restrictions in perceived participation.

### Isometric and isokinetic knee extension torque

Unilateral knee extension torque of either leg was assessed ~10 days before and ~3 days after completing the training intervention. The patient was positioned in the testing device (IsoMed2000 dynamometer; D&R Ferstl GmbH, Hemau, Germany; 200 Hz). Individual machine settings were recorded, saved and replicated in subsequent sessions. A standardized warm-up comprised three submaximal unilateral isometric actions at 90° knee flexion. Beginning with the non-affected leg, three maximal isometric actions, each sustained for 5 s, were executed 1 min apart. Peak torque (Nm) averaged over a 1-s window was chosen for data analysis. After 2 min recovery, unilateral CON and ECC isokinetic torque was assessed at 30, 60 and 90°/s, respectively. One min was allowed between different speed settings, and 2 min between CON and ECC actions. Three submaximal actions preceded each maximal attempt. The highest peak torque for each muscle action and speed mode was chosen for further analysis.

### Peak power

Peak power was assessed ~6 days before and ~5 days after the intervention. Patients completed 2 sets of 7 maximal CON-ECC unilateral actions for either limb using a flywheel leg press (YoYo® Technology AB, Stockholm, Sweden; Figure 1) device with a lateral leg support to avoid involuntary abduction of the affected limb during exercise. This apparatus provides unlimited resistance during coupled CON and ECC actions using the inertia of a spinning flywheel (0.036 kg · m^-2^) set in rotation by the trainee. Following initiation of flywheel momentum using modest effort, 7 consecutive repetitions were performed with maximal effort, accelerating rotation and hence speed of the wheel during CON, and produce deceleration in the subsequent ECC action. Patients were instructed to push with maximal effort through the entire range of motion in the CON action (i.e., from ~70° to almost full extension), then, and as the strap rewinds about the flywheel shaft, aim at resisting the inertial force. Thus, patients were requested to gently resist during the first third of the ECC action, and then apply maximal breaking force to stop the movement at about 70° knee flexion. Once the flywheel comes to a stop, a subsequent CON action is instantly initiated. This methodology and strategy has successfully been used to elicit ECC overload[20, 23]. Peak power was measured in all repetitions using an encoder (100 Hz) and associated software (SmartCoach™, Stockholm, Sweden). Three min recovery was allowed between sets. A 10-min warm-up on a cycle ergometer preceded the peak power tests.

### Isometric leg press force

Isometric LP force was measured using the flywheel device ~3 days before and ~7 days after the intervention. A foot-platform with a load cell was mounted for each foot on the exercise device, allowing individual force measurements (100 Hz) of each leg. Isometric tests were performed bilaterally and unilaterally for either leg at 90 and 120° knee angle. Patients were instructed to push as hard as possible for 5 s against the foot-platform, adjusted and fixed in the desired position using a chain system. Additional instructions and verbal encouragement to ensure maximal voluntary effort in tests using both the affected and non-affected limbs were offered. Two repetitions, with 1 min recovery in between, were carried out for each action mode. A third repetition was allowed if values differed >5%. Peak force averaged over a 1 s window was chosen for data analysis. A warm-up consisting of 10 min cycling and 3 submaximal bilateral isometric repetitions preceded these tests.

### Training intervention

Patients performed unilateral RE training using the affected leg on the flywheel LP device (Figure 1), 2 days per week during 8 weeks with ≥48 h of rest between sessions. Four sets of 7 repetitions at maximal effort were performed from ~70° knee flexion to almost full extension, with 3 min recovery between sets. Peak CON and ECC power was measured (see above) in all repetitions. Real time performance feedback was offered to the trainees at all times. Any training session followed a warm-up consisting of 10-min cycling at a submaximal load and one set of 7 coupled CON/ECC actions using modest effort on the LP apparatus.

### Data analysis

Results are presented as mean ± standard deviation (SD), unless otherwise indicated. Balance, gait and functional performance variables were analyzed by a one-way ANOVA over time. Isometric knee extension torque was analyzed using a two-way ANOVA with repeated measurements for time and leg. CON and ECC isokinetic torque were examined independently employing a three-way ANOVA (factors time, leg and speed). Peak power was analyzed independently for CON and ECC muscle actions by a two-way ANOVA with repeated measurements for time and leg. In addition, a two-way ANOVA (factors time x muscle action) was performed to assess differences between CON and ECC actions. Isometric LP force at 90 and 120° was examined separately using a three-way ANOVA with factors time, leg and mode (bilateral/unilateral). Training data were examined employing a two-way ANOVA (factors session and muscle action). Data normality was assessed through histograms and the Shapiro-Wilk test. When significant interactions were found, simple effect tests were employed. To compensate for multiple post hoc comparisons, the false discovery rate procedure was used[31]. The level of significant was set at 5% (P <0.05).

## Results

Fifteen patients, most of them being habitually active who recently had experienced infrequent low intensity strength training, were initially recruited. Medical issues, unrelated to the intervention *per se* prevented three individuals to complete the prescribed study protocol. Individual characteristics of the remaining 12 patients showing 100% compliance to the study protocol are depicted in Table 1. Six individuals were cognitively intact; 6 exhibited mild aphasia (5 showed expressive form and 1 individual combined expressive and impressive). Eleven patients were on medication i.e., antihypertensive, lipid lowering or anti-thrombotic, or combinations of these; 3 were prescribed anti-depressants i.e., selective serotonin reuptake inhibitors; 3 patients were on anti-epileptic drugs i.e., lamotrigin and levetiracetam or carbamazepine. Medication was neither altered nor introduced during the study. Four patients had received botullinum toxin injection at some time i.e., injection to arm and leg muscles 3 months (n = 1) or 24 months (n = 3) prior to the study.

**Table 1:** **Characteristics of the 12 patients that completed the study at baseline**

Patient	Age (yr)	Sex	Years since onset	Mechanism of stroke	Affected side	Walking aid
1	57.1	M	12.2	Hemorrhagic	R	None
2	57.8	F	5.9	Ischemic	L	Walking stick
3	62.3	M	17.2	Hemorrhagic	L	Forearm crutch
4	51.9	F	9.9	Ischemic	R	None
5	71.6	M	3.9	Ischemic	L	Walking stick
6	75.4	M	9.6	Ischemic	R	Walking stick
7	58.1	M	10.2	Hemorrhagic	R	None
8	66.2	M	3.0	Ischemic	L	None
9	69.3	F	3.9	Hemorrhagic	R	None
10	70.6	M	4.5	Ischemic	L	Rollator
11	68.6	M	2.4	Ischemic	R	None
12	50.7	M	10.9	Hemorrhagic	R	None
*Mean*	*63.3 ± 8.1*		*7.8 ± 4.5*			

### Training intervention

**Figure 2:**
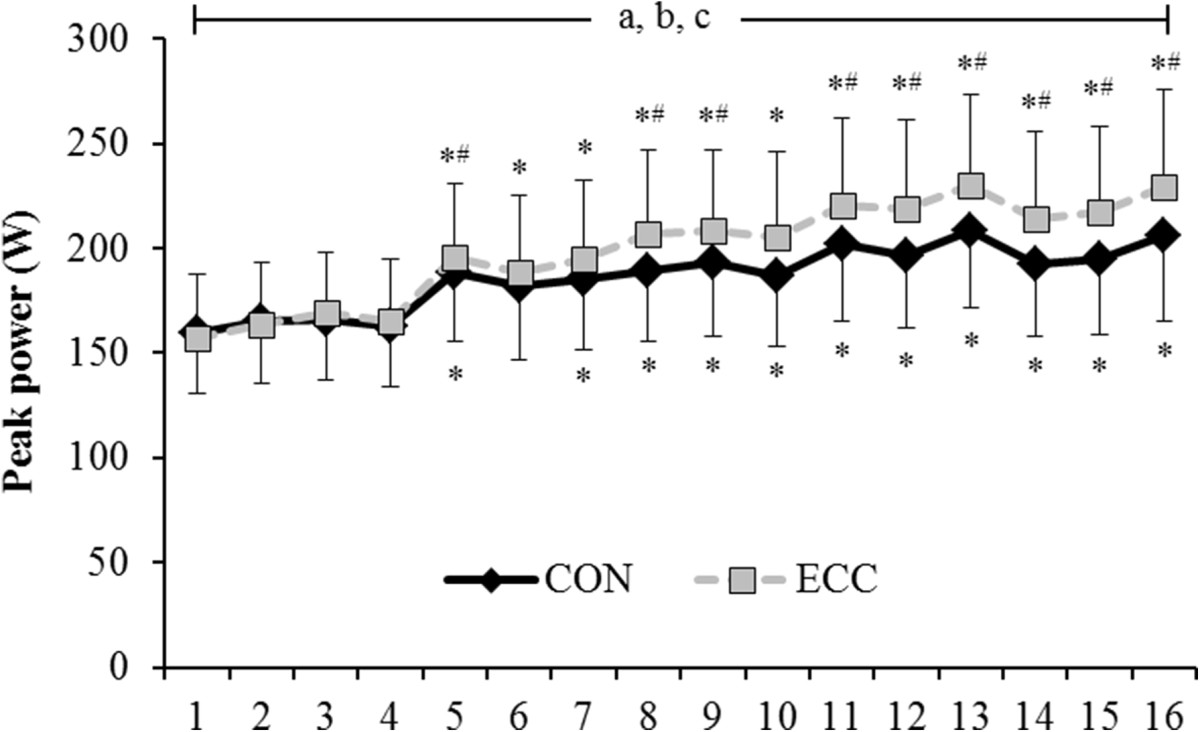
**Concentric (CON) and eccentric (ECC) leg press peak power (W) of the affected limb over 16 exercise sessions.** Significant main effects (P <0.05); a = interaction session x action, b = main effect of session, c = main effect of action. Significant simple effects (P <0.05); *vs. session 1; ^#^vs. CON action. Data presented as mean ± standard error of the mean.

There was a session x muscle action interaction for peak power (F = 5.5, P <0.0005; Figure 2). CON and ECC peak power increased from session 5 to 16 (P <0.05; except for CON in session 6; P = 0.082). Peak power was greater for ECC than CON in sessions 5, 8-9 and 11-16 (P <0.04).

### Isometric and isokinetic knee extension torque

The non-affected showed 1.4-fold greater isometric knee extension torque than the affected leg both pre and post training (Table 2; main effect of leg; F = 63.9, P <0.0005). There was no change over time for either leg. CON isokinetic peak torque was 1.3-fold greater in the non-affected than the affected leg (main effect of leg; F = 15.5, P = 0.002), and higher at low compared with high angular velocities (main effect of speed; F = 40.6, P <0.0005). CON isokinetic torque was unaltered after training (Table 2). ECC isokinetic peak torque showed leg x time interaction (F = 5.6, P = 0.038). Thus, only the affected, trained leg showed increased ECC-torque from pre to post training at 60 (8%; P = 0.036) and 90°/s (7%; P <0.0005; Table 2). Overall ECC torque was greater in the non-affected than the affected leg (main effect of leg; F = 56.1, P <0.0005).

**Table 2:** **Isometric and isokinetic knee extension torque (Nm) pre and post training**

	Affected leg			Non-affected leg		
	Pre	Post	Δ%	Pre	Post	Δ%
Isometric torque^b^	135 ± 34	138 ± 39	2	195 ± 41^#^	187 ± 35^#^	-4
CON torque at 30°/s^b, c^	113 ± 29	120 ± 32	6	147 ± 33^#^	144 ± 37^#^	-2
CON torque at 60°/s^b, c^	99 ± 28	102 ± 32	3	123 ± 42^#^	131 ± 40^#^	6
CON torque at 90°/s^b, c^	86 ± 26	88 ± 34	2	111 ± 46^#^	115 ± 50^#^	4
ECC torque at 30°/s^b^	144 ± 40	149 ± 40	3	171 ± 36^#^	172 ± 35^#^	1
ECC torque at 60°/s^a b^	143 ± 41	154 ± 45*	8	178 ± 40^#^	175 ± 37^#^	-2
ECC torque at 90°/s^a b^	141 ± 36	151 ± 39*	7	173 ± 36^#^	174 ± 36^#^	1

CON; concentric, ECC; eccentric. Significant main effects (P <0.05); ^a^interaction leg x time; ^b^main effect of leg; ^c^main effect of speed; Significant simple effects (P <0.05); *vs. pre value within a leg; ^#^vs. affected leg for a time point.

### Peak power

**Figure 3:**
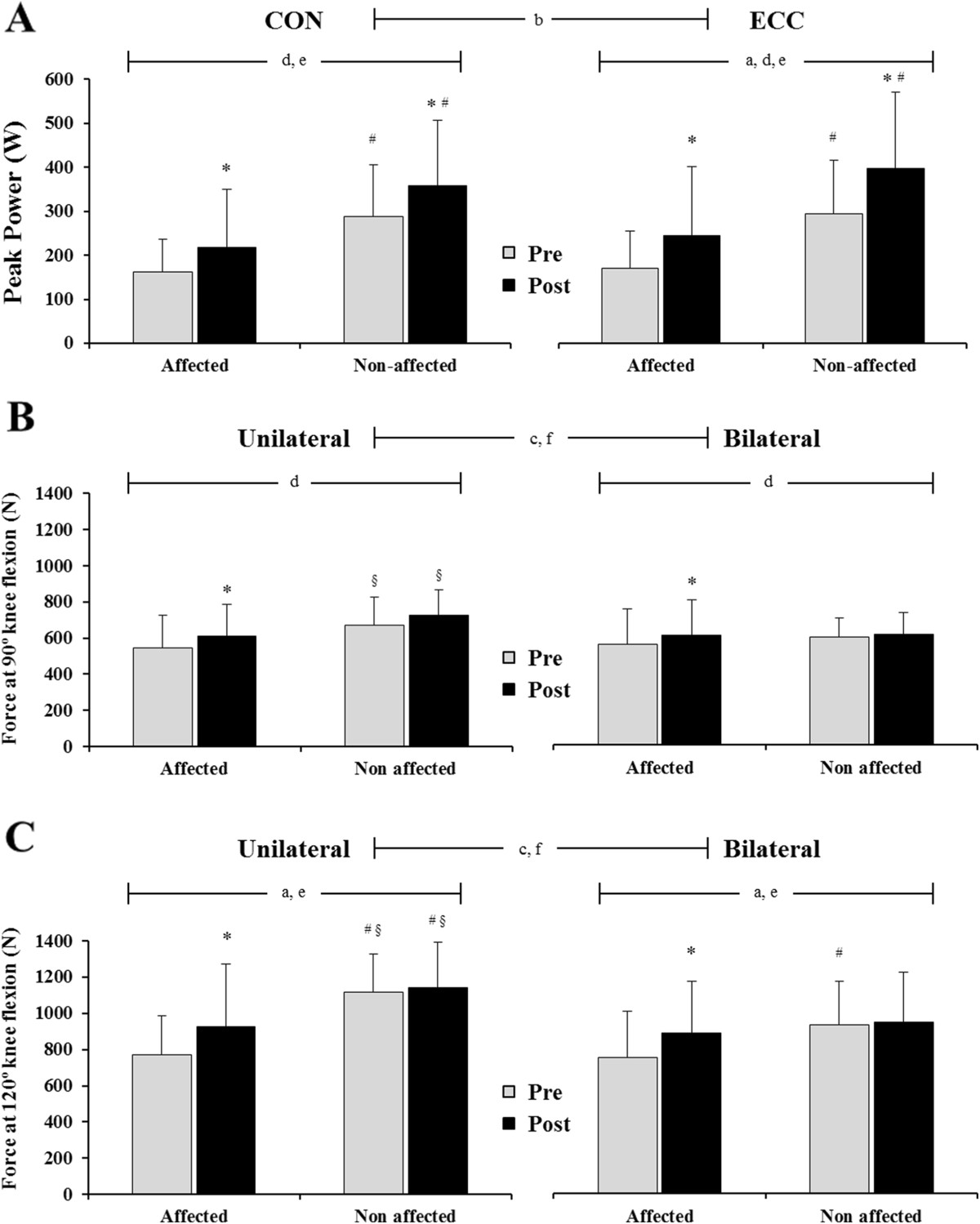
**Concentric (CON) and eccentric (ECC) leg press peak power (W) of the affected and non-affected limbs (A), and isometric force in the leg press at 90° (B) and 120° (C) knee angle for the affected and non-affected leg during unilateral and bilateral tests performed pre and post training.** Significant main effects (P <0.05); a = interaction time x leg, b = interaction time x muscle action, c = interaction leg x mode, d = main effect of time, e = main effect of leg, f = main effect of mode. Significant simple effects (P <0.05); *vs. Pre within a leg; ^#^vs. affected leg; ^§^vs. bilateral mode within a leg and time point.

There was a time x muscle action interaction (F = 11.0, P = 0.008) in peak power. Thus, ECC peak power increased more than CON in both the affected and the non-affected leg (Figure 3A). There was a time x leg interaction in ECC peak power (F = 5.1, P = 0.046). Thus, although ECC peak power increased in both the affected and non-affected leg (affected; P = 0.019; non-affected; P = 0.003), the gains were greater for the affected leg (44% vs. 35%). In addition, the non-affected leg produced more ECC peak power both pre (P = 0.001) and post training (P <0.0005), compared with the affected limb. CON peak power showed no time x leg interaction. However, there was a main effect of time (F = 10.2, P = 0.009) as CON peak power increased in both the affected (34%) and the non-affected (24%) leg. In addition, there was a main effect of leg (F = 31.7, P <0.0005) due to greater overall CON peak power in the non-affected than the affected leg.

### Isometric leg press force

Isometric LP force at 90° knee angle. There was a main effect of time (F = 5.1, P = 0.045) mainly as the affected leg produced greater force at post compared with pre training (P = 0.003 and P = 0.008 for unilateral and bilateral mode, respectively). There was a leg x mode interaction (F = 9.4, P = 0,011; Figure 3B). Thus, while the affected leg produced similar force during bilateral and unilateral conditions, the non-affected leg produced higher force in the unilateral than the bilateral test, both at pre (P = 0.036) and post (P = 0.001) training.Isometric LP force at 120° knee angle. There was a time x leg interaction (F = 8.6, P = 0.014; Figure 3C). Thus, while the affected leg showed increased isometric force both bilaterally (17%, P = 0.045) and unilaterally (20%, P = 0.021), force of the non-affected leg remained unchanged. Force was greater for the non-affected than the affected leg at pre training in both the bilateral (P = 0.027) and unilateral (P <0.0005) mode. Force was greater in the affected vs. non-affected limb in the unilateral mode only at post training (P = 0.025). There was a leg x mode interaction (F = 10.7, P = 0.008). Thus, the non-affected leg produced more force in unilateral than bilateral modes at pre (P = 0.01) and post (P = 0.004) training. Force of the affected leg was similar across modes.

### Balance, gait, functional performance, spasticity and perceived participation

Balance (BBS, 7%, P = 0.004), TUG (17%, P = 0.018) and 30CST (17%, P = 0.024) improved with training (Table 3). There were no changes in 6MWT distance (P = 0.68) or Modified Ashworth Scale score (P = 0.24; Table 3). There was an increase in perceived participation (SIS) after training that was at the limit of statistical significance (P = 0.058).

**Table 3:** **Balance, gait, functional performance and perceived participation pre and post training**

	Pre	Post
Berg Balance Scale (a.u.)	48.5 ± 8.7	51.7 ± 6.4*
Timed Up-and-Go (s)	16.9 ± 9.1	14.1 ± 7.3*
6-minute Walk Test (m)	292.9 ± 144.5	295.3 ± 146.8
30-second Chair-Stand (reps)	8.5 ± 3.5	9.9 ± 4.6*
Modified Ashworth Scale (a.u.)	0.77 ± 0.54	0.88 ± 0.55
Stroke Impact Scale (a.u.)	62.2 ± 14.5	66.0 ± 12.8^§^

a.u.: arbitrary units, s: seconds, m: meter, reps: repetitions. Significant differences: *vs. Pre (P <0.05); ^§^at the limit of statistical significance vs. Pre (P = 0.058).

## Discussion

The current study assessed the efficacy of a novel ECC-overload flywheel RE training paradigm to improve force, power, balance and functional performance in physically active chronic stroke patients (2-17 years post stroke). Patients complying with the 8-week intervention showed marked gains in muscle power of the trained affected, but of the untrained non-affected limb as well. Also, there were increases in isometric leg press force and isokinetic ECC, not CON, knee extension torque, accompanied by improved balance and functional performance without exaggerating spasticity. Thus, the results of this investigation suggest that short-term ECC-overload flywheel RE training is a valid, safe and viable method to improve muscle function, balance, gait and functional performance in men and women suffering from stroke.

The ECC-overload flywheel RE training was well received by chronic patients, as indicated by the overall 37% increase in peak power across sessions (Figure 2), without exacerbating spasticity. Despite the low volume or exercise dose carried out in each training session, i.e., 28 coupled CON-ECC actions equivalent to <1 min of contractile activity, power and balance improvements induced by the current intervention were robust and comparable to those achieved after more extended (i.e., 12 weeks) training[11, 32] using conservative methods employing either isokinetic or gravity dependent loading. Thus, unique features characteristic of the exercise training paradigm used here and elsewhere[19–24], allowing for brief episodes of ECC-overload, unrestricted CON force of any action through the entire range of motion, variable velocity, and call for acceleration and deceleration of each coupled CON-ECC action, appear to reinforce the positive effects of RE, beyond what has been noted in stroke patients subjected to more conservative RE methods[11, 32].

As muscle power, more so than strength, correlates with physical performance in individuals showing restricted mobility[33, 34], rehabilitation programs prescribed to stroke patients with obvious gait deficits, should target muscle power as a primary outcome. Interestingly, the marked increase in peak power of the trained affected leg after the current intervention was accompanied by an increase of nearly the same magnitude in the untrained non-affected leg. Such cross-education effect has previously been reported in stroke patients subjected to RE employing ECC actions only[17], inferring RE favoring ECC actions prompts important central nervous system adaptations[35]. In support, cortical activity is greater during ECC than CON actions[36]. Hence, neural signaling evoked by ECC exercise, and perhaps ECC-overload flywheel RE even more so, may reinforce certain neural strategies due to the variable velocity, and accelerating and decelerating coupled CON-ECC muscle actions, executed at maximal effort[37]. In support, ECC-overload flywheel RE elicited more prominent neural adaptations than constant load CON-ECC RE training in healthy individuals[24]. Altogether, the specific features of the current exercise modality appear to facilitate more favorable stimulus than traditional constant load or velocity (i.e., isokinetic) RE training paradigms, because of the emphasis on stretch-shortening cycle and stretch reflex, resulting in increased afferent traffic and proprioception, and/or the unique motor unit recruitment strategy typical of ECC actions. Indeed, the current resistance exercise paradigm seems to induce greater cross-education adaptations than more traditional training protocols[17, 38].

Isometric leg press force was higher, and increases in response to training greater, at 120 than 90° knee angle, suggesting stroke patients not only exhibit more weakness in the innermost part of the range of motion of a joint[4], but are also less prone to benefit from the exercise stimulus imposed in that particular range of motion. The overall increase in leg press force for the paretic limb amounted to 10-20%. Neither limb showed increased isometric knee extension torque post training. This finding concerts the principle of training specificity suggesting the transfer effect is most evident in functional mode(s) mimicking the particular exercise executed during training[39].

Bilateral maximal voluntary force deficits, such that the summed force produced by each limb alone exceeds force in a bilateral action, are well documented in healthy individuals. In the current investigation, bilateral asymmetry was evident for the non-affected leg (<15% force in the bilateral action), both pre and post training, and regardless of knee angle. This observation is consistent with the report of McQuade et al.[40], noting bilateral asymmetries in the non-paretic *m.* biceps brachii of stroke patients executing isometric elbow flexions, yet contrasts reports showing bilateral asymmetries of the affected leg only[41] or both legs[42], when subjected to lower limb isometric actions. While ample evidence suggests neural factors are responsible for these deficits[43, 44], inconsistencies in outcome across the above studies remain to be explored, e.g., time since stroke, severity of impairment, and muscle specific recruitment of neural pathways.

Balance, a critical asset in any upright position with obvious impact on quality of life[45, 46] and physical performance in daily activities[47], is typically impaired after stroke. The current low-volume, high-load RE protocol induced early significantly enhanced balance, assessed by means of BBS. Yet, only one patient attained the proposed limit for minimal clinically significant difference for the elderly, i.e., an 8-point increment[48]. It is worth noting that patients presenting lower BBS scores before training experienced the most substantial improvement in balance. Thus, while five patients displayed scores ≤45 (range 28-45), indicating increased risk of fall[49] prior to training, only one patient appeared to be at risk after the intervention (BBS score 34). Even though the current method assessing balance, i.e., BBS, may have allowed for a “ceiling effect” in some patients, it remains the novel exercise intervention employed here, improved balance in stroke patients.

The current training paradigm also improved 30CST and TUG performance. Changes in 30CST between 2.0 and 2.6 are associated with improvements corresponding to minimum clinically important difference[50]. In the present study, increases between 2 and 6 suggest 6 patients exhibited clinically important improvements. TUG scores between 0.8 and 1.4 sec infer major improvement[50]. In the present study, 9 patients showed enhanced TUG performance by 1 to 13 sec, indicating major significant clinical improvement.

Walking distance, as reflected in the 6MWT, was unchanged. While our exercise regime did not intend to improve walking, some reports have inferred a causal relationship between muscle strength and walking capacity[51, 52], or that RE may serve to improve comfortable gait speed and total distance walked[53]. It is worth recalling that whereas use of the hip flexors is critical for walking in stroke patients[51], involvement of this muscle group was not emphasized with the current RE paradigm, which rather called for closed-chain, simultaneous knee- and hip extension. Further, given the particular exercise stimulus imposed in this investigation (<2 min per week), patients were not anticipated to benefit in the 6MWT. Nevertheless, the RE paradigm employed here enhanced TUG, an established mean assessing gait performance after stroke[13, 14]. Thus, while features related to short-distance walking, like fastest self-selected speed were improved after ECC-overload RE training, additional exercise tasks are warranted to carry on walking over longer distances.

Conclusions from the current pilot study should consider that individuals examined comprised 12 chronic, yet low severity disabled stroke patients with minimal spasticity. Future research employing ECC-overload flywheel RE warrants investigations comprising more severely injured stroke victims, as well as control individuals, to affirm the applicability of this novel exercise rehabilitation paradigm to a broader range of men and women suffering from stroke.

## Conclusions

The current 8-week flywheel RE paradigm, prescribed to stroke patients, facilitated robust increases in muscle strength and power of the affected trained limb, as well as improved power of the non-affected untrained leg. These adaptations were accompanied by significantly enhanced balance, gait and functional performance. While this particular iso-inertial exercise insult allows for unrestricted force through the entire range of motion of any performed CON muscle action, it also offers brief episodes of ECC-overload. Our novel exercise paradigm appears to present a safe, viable and highly effective method to improve skeletal muscle function, and performance in daily living activities, in individuals suffering from chronic stroke.

## Authors’ original submitted files for images

Below are the links to the authors’ original submitted files for images. Authors’ original file for figure 1 Authors’ original file for figure 2 Authors’ original file for figure 3
