# Supplementary figures and images for: Chronic stroke patients show early and robust improvements in muscle and functional performance in response to eccentric-overload flywheel resistance training: a pilot study
Source: J Neuroeng Rehabil. 2014 Oct 30;11:150. doi: 10.1186/1743-0003-11-150 (PMC4236468; doi:10.1186/1743-0003-11-150)

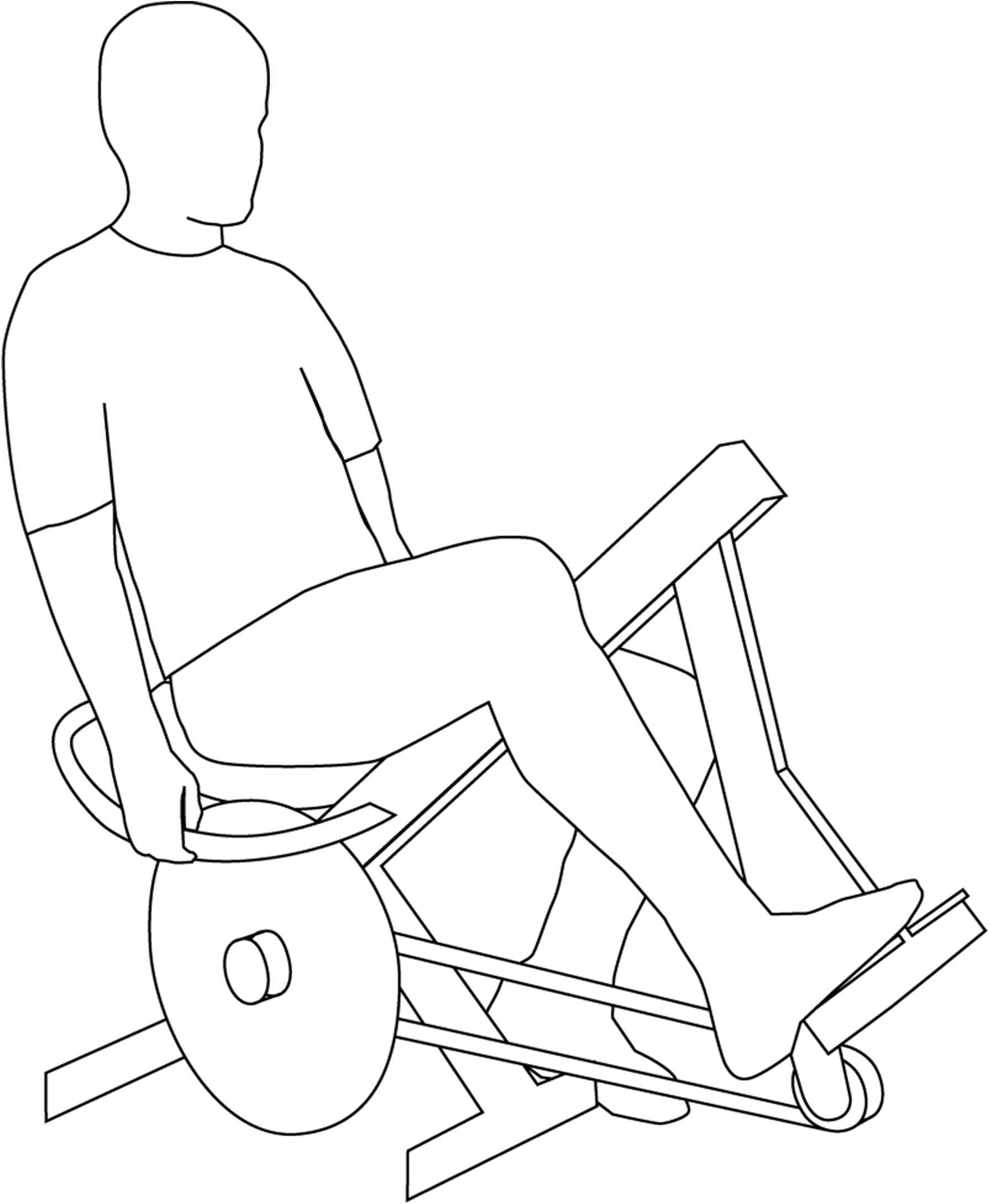

Supplement: Supplementary file 1 — Authors’ original file for figure 1 [file 12984_2014_667_MOESM1_ESM.tif]

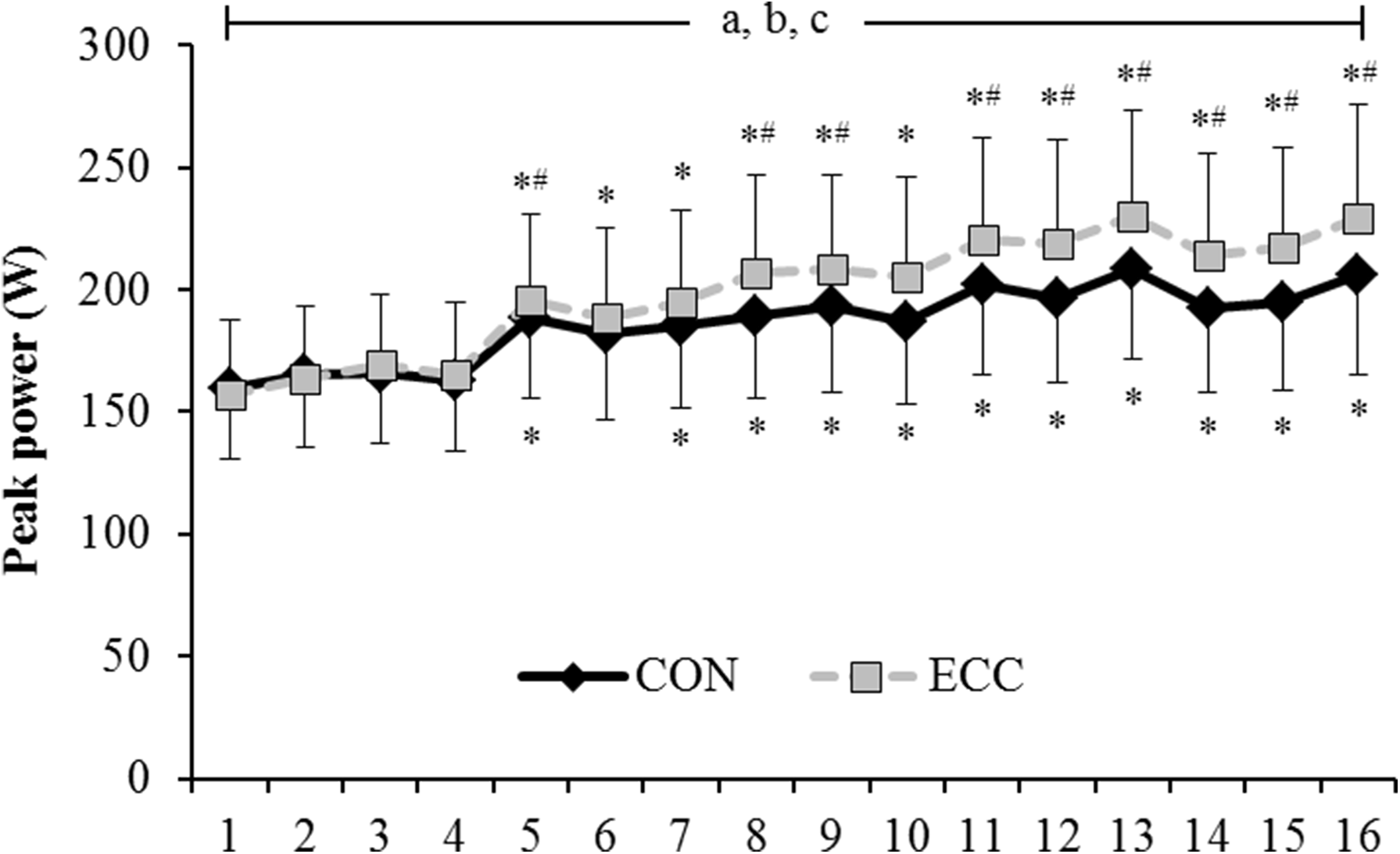

Supplement: Supplementary file 2 — Authors’ original file for figure 2 [file 12984_2014_667_MOESM2_ESM.tif]

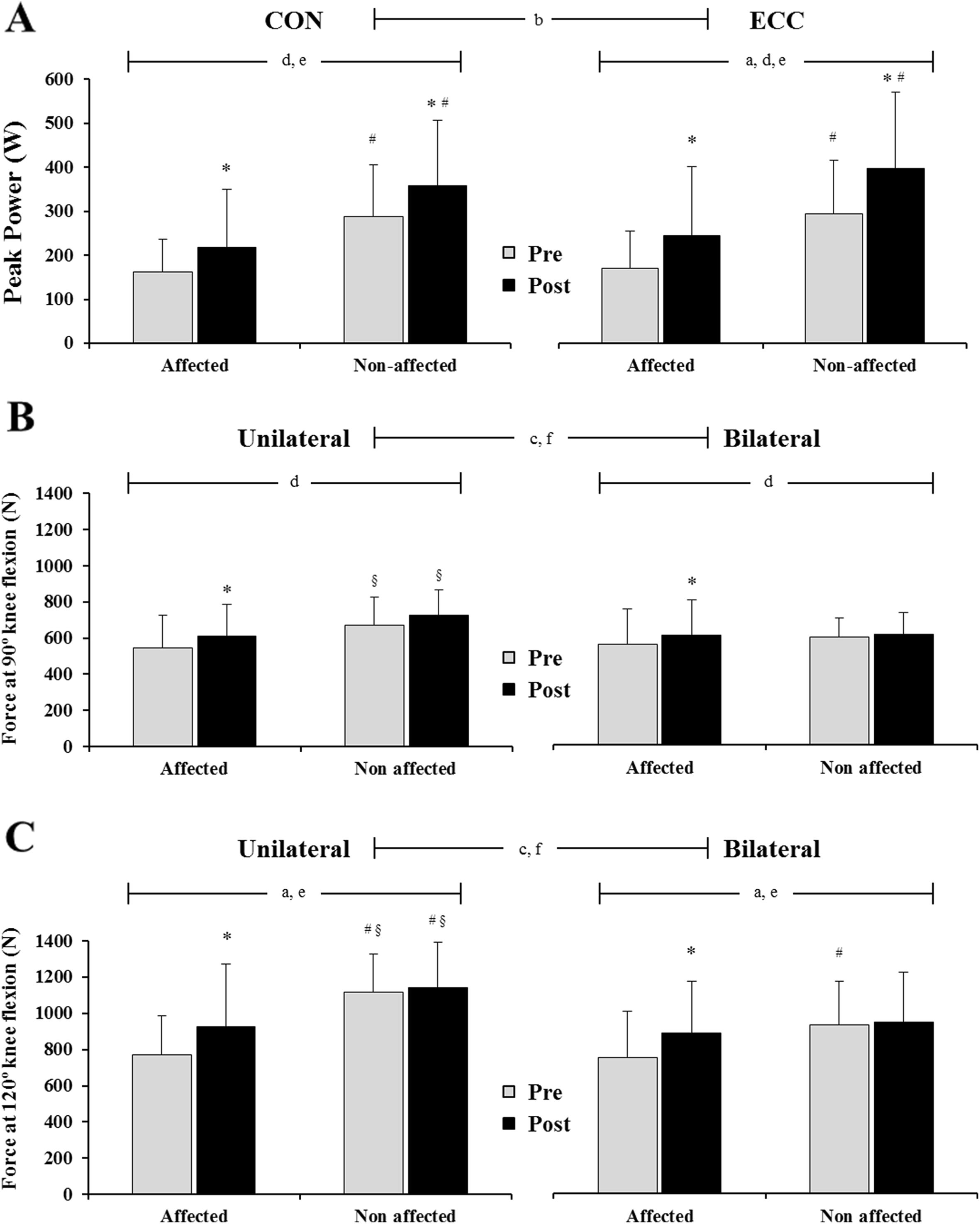

Supplement: Supplementary file 3 — Authors’ original file for figure 3 [file 12984_2014_667_MOESM3_ESM.tif]
